# A Comparative Study on the Characteristics of Different Types of Camellia Oils Based on Triacylglycerol Species, Bioactive Components, Volatile Compounds, and Antioxidant Activity

**DOI:** 10.3390/foods13182894

**Published:** 2024-09-12

**Authors:** Beibei Duan, Hoe-Seng Tin, Chengwen Mao, Xing Tong, Xuehui Wu

**Affiliations:** 1 College of Food Science, South China Agricultural University, Guangzhou 510642, China; bbduan@outlook.com; 2 Foshan Haitian Flavouring and Food Co., Ltd., Foshan 528000, China; 3 One Healthin Technology Co., Ltd., Foshan 528200, China; 4 Guangdong Haitian Innovation Technology Co., Ltd., Foshan 528000, China; 5 Key Laboratory of Advanced Technology Enterprise of Guangdong Seasoning Food Biofermentation, Foshan 528000, China

**Keywords:** camellia oils, camellia diacylglycerol oil, triacylglycerol species, bioactive components, volatile compounds, antioxidant activity

## Abstract

This study aimed to investigate the characteristics of different varieties of camellia oils and their diacylglycerol (DAG)-enriched derivatives in terms of triacylglycerol (TAG) species, bioactive components, volatile compounds, and antioxidant activity. Six types of camellia oils, including *C. oleifera* (C.O), *C. semiserrata* (C.S), *C. gauchowensis* (C.G), along with commercially refined *C. oleifera* oil (C-C.O) and its DAG-enriched counterparts (at 40% and 80% enrichment), were analyzed and compared. Unique patterns of TAG profiles, fatty acid distributions on different glycerol backbones, tocopherol, squalene, total polyphenols, and volatile compounds were observed, suggesting that these characteristics can be utilized as a criterion to differentiate them. DAG-enriched oils exhibited increased levels of unsaturated fatty acids (UFAs) compared to C-C.O, albeit with decreased contents of tocopherol, squalene, and total polyphenols. Moreover, diverse volatile compounds were identified across all types of camellia oils, among which the DAG-enriched oils had distinct distribution characteristics compared with their crude oils, indicating the influence of the enrichment process on volatile compounds. Furthermore, DAG-enriched oils demonstrated reduced antioxidant activity abilities compared to their counterparts, with the highest activity observed in C.O, followed by C.G. Additionally, strong correlations were observed between antioxidant activity and tocopherol, as well as squalene content.

## 1. Introduction

Camellia oil, derived from the seeds of the *Camellia oleifera* plant, stands as a versatile and culturally significant substance with a rich history spanning centuries [1]. This edible vegetable oil has found its roots in Asian culinary traditions, particularly in China and Japan, where it has been celebrated for its health benefits and numerous applications [2,3].

A large number of studies have garnered attention into the potential health benefits of camellia oil, which have demonstrated numerous functions, such as preventing cardiovascular disease [4,5], inhibiting tumor growth [6], and preventing Alzheimer’s disease [7,8]. Additionally, researches have highlighted the essential roles of camellia oil in anti-asthmatic, anti-diabetic, anti-inflammatory, antioxidant, and antibacterial aspects [9,10,11]. Considering the various nutritional functions of camellia oil, its composition has been meticulously analyzed, with a particular emphasis on identifying and understanding the bioactive components.

Various bioactive components in camellia oil have been reported, including fatty acids, tocopherol, squalene, polyphenols, and volatile compounds, etc. [2,3]. As for the fatty acids of camellia oil, oleic acid stands out as the most abundant, constituting approximately 60.20–84.80%, followed by palmitic acid (7.10–31.30%) and linoleic acid (4.10–10.10%) [12,13,14]. Given this composition, the levels of unsaturated fatty acids (UFAs) in camellia oil typically range from 65.80% to 89.50%, which is similar to olive oil, resulting in camellia oil being known as the Oriental olive oil [12,13,14,15].

In addition, the molecular species of triglycerides (TAGs) in camellia oil were studied, which are formed by connecting three fatty acid residues to one glycerol molecule. Given that oleic acid is the most abundant fatty acid in camellia oil, it was anticipated that the predominant TAG in camellia oil would be triolein (OOO), accounting for 45.43–47.69%, followed by palmitic–oleic–oleic (POO; 14.36–17.81%), together accounting for over 60% of total TAGs. Additionally, small amounts of TAGs, such as oleic–oleic–linoleic (OOL; 6.62–13.43%), palmitic–oleic–linoleic (POL; 3.19–5.59%), and palmitic–palmitic–oleic (PPO; 1.79–4.05%), were also observed in camellia oil [16,17].

As a natural fat-soluble antioxidant, tocopherol prevents lipid oxidation and reduces the degree of peroxidation in cells [18], a property also observed in camellia oil. According to the literature [16,19,20], the content of α-, γ-, and δ-tocopherol in camellia oil was 3.60–771.00, 9.40–59.00, and 0.27–28.00 mg/kg, respectively. Notably, β-tocopherol was not detected in camellia oil. Additionally, squalene, an intermediate in the biosynthesis of phytosterols or cholesterol in plants and humans [21,22], was found in camellia oil, with a concentration ranging from 26.33 to 756.38 mg/kg [16,20,23]. Furthermore, the functions of squalene in anti-cancer, antioxidant, and other biological functions have been confirmed [24,25]. Similar to squalene, polyphenols exhibit a range of biological activities, such as anti-inflammatory, antioxidant, anti-tumor, and cardiovascular regulation [10,26]. Wang et al. [23] reported the polyphenol content in camellia seed oil from six different areas, with the total polyphenol content ranging from 43.32 μg/g to 72.77 μg/g. Additionally, volatile compounds, used as an index to evaluate the quality of camellia oil, have been documented in some studies [27,28], wherein the presence of aldehydes (17.03–44.54%), hydrocarbon (10.35–58.98%), alcohol (3.15–31.71%), ketones (1.12%), and esters (3.93–8.15%) was revealed.

Various varieties of camellia oils, such as *C. oleifera, C. meiocarpa*, *C. vietnamensis*, *C. yuhsienensis*, *C. chekiangoleosa*, *C. semiserrata*, and *C. gauchowensis* currently are mass-produced in China. Among these, *C. oleifera* holds the largest cultivated acreage, accounting for 98% of the total [29]. Different varieties of camellia oil may exhibit variations in their triacylglycerol (TAG) species, bioactive components, and volatile compounds. Wei et al. [17] and Shi et al. [30] demonstrated that TAG and squalene can be valuable markers for characterizing different edible vegetable oils, including soybean oil, camellia oil, palm oil, coconut oil, flaxseed oil, and pine seed oil, etc. Unfortunately, limited research has focused on distinguishing between different varieties of camellia oil, with only Tang et al. [31] reporting that bioactive components could distinguish camellia oil varieties. However, even for the same variety of camellia oil, differences in the growth environment, processing technology, and analysis methods may lead to differences in the characteristics of camellia oil. Commercial camellia oils often undergo complex manufacturing processes, resulting in differences from crude camellia oil. Therefore, further systematic investigations and comparisons of the ingredients in different varieties of camellia oils are necessary to identify markers that can distinguish their origin.

Recently, there has been growing interest in diacylglycerol (DAG)-enriched camellia oils, which stems not only from the rich nutritional and functional properties of camellia oil but also concerns about the increasing prevalence of obesity and various metabolic syndromes associated with excessive fat intake. As a natural oil component, DAG is found in various plant oils, with a maximum content of 10% [32]. Compared to TAG, although DAG and TAG are digested by the same gastrointestinal enzymes, 1,3-DAG follows a distinct pathway. After digestion, 1,3-DAG does not undergo the 2-monoacylglycerol (2-MAG) pathway and glycerol-3-phosphate pathway of TAG, preventing the resynthesis of new TAG [33]. This unique characteristic has shown that DAG offers better benefits than TAG in clinical studies, emerging as a potential and healthier alternative to traditional edible TAG oil. For instance, DAG promotes fat oxidation, inhibits weight gain, reduces visceral fat, and improves cardiovascular and cerebrovascular health [34].

As the low content of DAG was observed in plant oil, numerous studies have been carried out on the bio-enzymatic synthesis of DAG-enriched oils using vegetable oil or glycerol and fatty acids as substrates. Among these, the esterification method, glycerol hydrolysis method, and enzymatic hydrolysis method were mainly used [34]. A comparative study on the physicochemical properties of DAG-enriched oil after synthesis revealed that the viscosity and specific gravity of DAG-enriched oil are slightly higher than those of TAG. Additionally, the smoke, flash, fire, and ignition points of DAG-enriched oil are 30–40 °C lower than those of TAG oil [35]. Unfortunately, limited information is available regarding changes in its bioactive components, volatile compounds, and antioxidant activity, which are important aspects for evaluating the functionality of DAG-enriched oils for applications. Therefore, a more systematic understanding of DAG-enriched oil requires an exploration of the differences between TAG and DAG-enriched oil in these aspects.

To better understand and distinguish different camellia oils and their DAG-enriched derivatives, this study analyzed three camellia oil varieties (*C. oleifera*, *C. semiserrata*, and *C. gauchowensis*), along with a commercially refined *C. oleifera* oil and its DAG-enriched variants. Their TAG species, bioactive components (fatty acids, tocopherols, squalene, polyphenols), volatile compounds, and antioxidant activity were examined. The results were used to identify key markers for differentiating among these oils. 

## 2. Materials and Methods

### 2.1. Materials and Chemicals

Triundecanoin (C11:0), BF_3_-methanol (14%, *w*/*w*), and standards of fatty acid methyl esters (Supelco 37 components FAME mix), tristerarin, glyceryl disterarate, stearic acid, α-tocopherol, rac-β-tocopherol, γ-tocopherol, δ-tocopherol, and squalene were purchased from Sigma–Aldrich Chemical Co., Ltd. (Shanghai, China). Kits for the ferric reducing antioxidant power (FRAP) and 1, 1-diphenyl-2-picrylhydrazyl (DPPH) and 3-ethylbenzothiazoline-6-sulfonicacid (ABTS) radical scavenging activity were acquired from Suzhou Grace Biotechnology Co., Ltd. (Suzhou, China). Solvents and chemicals used in this study were purchased from Shanghai Titan Scientific Co., Ltd. (Shanghai, China). The solvents used in this study were all analytical grade unless otherwise stated.

Different varieties of camellia oil are cultivated at the Zengcheng Teaching and Research Base of South China Agricultural University (113.63° N, 23.23° E), situated in a typical southern subtropical monsoon climate zone characterized by moderate temperatures, abundant rainfall, and ample sunlight. Dried camellia seeds of three varieties, namely *C. oleifera* (C.O), *C. semiserrata* (C.S), and *C. gauchowensis* (C.G), were provided by the College of Food Science, South China Agricultural University (Guangzhou, China). After dehulling, the seeds underwent oil extraction using a screw press at temperatures ranging from 60 to 90 °C. The extracted camellia oil was then obtained through centrifugation to remove impurities. Commercially refined *C. oleifera* oil (C-C.O) oil obtained through low-temperature cold pressing (60–90 °C) without any additives was purchased from Zhejiang Choisun Tea Development Co., Ltd (Hangzhou, China). Diacylglycerol (DAG)-enriched camellia oils (40% and 80%) were prepared using an enzymatic method described by Prabhavathi Devi et al. [36], using the commercial *C. oleifera* oil (C-C.O) as the raw material. Briefly, 250 g of C.O. and 150 g of distilled water were mixed, and 2% of Lipozyme TLIM lipase was added for hydrolysis. The reaction was conducted on a shaking table at 40 °C for 24 h, followed by molecular distillation to obtain free fatty acids. These free fatty acids were then reacted with glycerol at a molar ratio of 1:0.75, using 5% of 1,3-specific lipase Lipozyme RMIM. The reaction was performed in a reactor at 60 °C with a stirring speed of 500 rpm for 5–8 h. Finally, the DAG-enriched camellia oil, containing a mixture of 1,2- (and 2,3-) isomers and 1,3-isomers, was obtained after molecular distillation of the esterification product. DAG-enriched oils with a purity of 40% and 80% were named C-C.O-DAG (40%) and C-C.O-DAG (80%), respectively.

### 2.2. Glyceride Distribution Analysis

Glyceride distribution analysis was conducted in accordance with the Chinese Standard GB/T 26636-2011 [37], with modifications. To analyze the distribution of glycerides in oil samples, an Agilent 1260 Infinity II LC (Santa Clara, CA, USA) equipped with an Agilent PL gel MIXED-E column (7.5 mm × 300 mm, 3 µm, Santa Clara, CA, USA) and an Agilent 1260 Infinity Refractive Index Detector (Santa Clara, CA, USA) was used. Tetrahydrofuran was employed as the mobile phase for isocratic elution at a flow rate of 0.5 mL/min. The oil sample was diluted with tetrahydrofuran to a concentration of 15 mg/mL, with an injection volume of 20 μL, and the total analysis time was 20 min (Figure S1).

### 2.3. Triacylglycerol Species Analysis

Triacylglycerol (TAG) species analysis was performed according to the method reported by Wei et al. [17] with some modifications. An UHPLC-Q-TOF-MS (Agilent, 1290 Infinite II-6545B) equipped with a ZORBAX Eclipse Plus C18 column (50 mm × 2.1 mm, 1.8 μm; Agilent, Palo Alto, CA, USA) was utilized. The column temperature was set at 35 °C, and the flow rate was 0.3 mL/min. A total of 30 mg of each oil sample was weighed and diluted to 1 mg/mL with acetonitrile, and the injection volume was 2 μL. Mobile phase A consisted of acetonitrile and ammonium formate (10 mmol/L) in a ratio of 98:2 (*v*/*v*), and mobile phase B comprised isopropanol and ammonium formate (10 mmol/L) in a ratio of 98:2 (*v*/*v*). The gradient elution proceeded as follows: mobile phase A was 60% for 0–2 min, 50% for 2–5 min, 40% for 5–10 min, and 60% for 10–15 min.

Mass spectrometry equipped with the electrospray ion source (ESI) was employed, with the following conditions: the positive ion mode was used as the scan mode within a scanning range of 100–1700 *m*/*z*. High-purity nitrogen served as the curtain gas, atomizing gas, and collision gas. The curtain gas operated at a temperature of 320 °C with a flow rate of 8.0 L/min, and the sheath gas temperature was 350 °C with a flow rate of 11.0 L/min. The ion spray voltage was set at 4000 V. Data acquisition and analysis were performed using Data Acquisition (Agilent, Palo Alto, CA, USA) and Q-TOF Quantitative Analysis (Agilent, Palo Alto, CA, USA), respectively. The TAGs in the oil samples were identified using positive-ion ESI-MS by detecting protonated molecules [M+NH_4_]^+^ and fragment ions [M+H-RCOOH]^+^, which facilitated the assignment of TAG molecular structures and the identification of individual fatty acids.

### 2.4. Bioactive Component Analysis

#### 2.4.1. Fatty Acid Analysis

The methyl esterification of oil samples was required before analyzing total fatty acids [38]. Briefly, 0.1 g of the oil sample was accurately weighed into a 100 mL round-bottomed flask. Subsequently, 2 mL of triundecanoin (C11:0, internal standard) and 8 mL of 2% sodium hydroxide solution (in methanol) were added. The flask was then stirred in a water bath at 80 °C with condensation and reflux for 2 h. Afterward, 7 mL of BF_3_-methanol (15%, *w*/*w*) was added to continue the reaction for 2 min. The flask was taken out after cooling with water until cloudy, and 20 mL of n-heptane and 10 mL of saturated sodium chloride solution were added. The mixtures were stirred for 2 min, and the upper organic phase was taken for GC analysis after standing for stratification.

Fatty acids were separated using an Agilent 8890 Gas Chromatograph (Santa Clara, CA, USA) fitted with a flame ionization detector (FID) and a CP-Sil 88 capillary column (100 m × 0.25 mm, 0.20 μm; Chrompack, Varian Inc., Walnut Creek, CA, USA). Nitrogen was used as the carrier gas at a flow rate of 1.0 mL/min. The split ratio was 100:1, and the injector and detector temperatures were set at 270 °C and 280 °C, respectively. The oven conditions were as follows: 100 °C held on for 13 min, then increased to 180 °C at a rate of 10 °C/min, and held for 6 min; subsequently raised to 200 °C at a rate of 1 °C/min, and held for 20 min; finally increased to 225 °C at a rate of 4 °C/min, and held for 12 min.

The sn-2 positional fatty acid compositions of the oil samples were determined using pancreatic lipase following the procedure outlined by Lee at al. [38]. In brief, 20 mg of the oil sample was combined with 20 mg of pancreatic lipase. The mixture was then supplemented with 7 mL of 0.1 mM Tris-HCl buffer (pH 7.6), 0.7 mL of 2.2% calcium chloride, and 0.7 mL of 0.05% bile salt. Subsequently, the mixture was incubated in a 37 °C water bath for 3 min and then vortexed for 30 s; this cycle was repeated three times. Afterward, 4 mL of diethyl ether was added to the mixture and centrifuged at 2500 rpm for 5 min after vortexing for 1 min. The supernatant was collected through an anhydrous sodium sulfate column for loading onto thin-layer chromatography (TLC). A mixture solution of diethyl ether:hexane:acetic acid (50/50/1, *v*/*v*/*v*) was used as the development solvent. The isomerization of 2-monoacylglycerols to 1(3)-monoacylglycerols on silica gel TLC can occur due to the slightly acidic surface of the silica gel, caused by the presence of silanol (Si-OH) groups. Therefore, the monoacylglycerol band was scraped off and methylated for GC analysis, as described above.

#### 2.4.2. Tocopherol Analysis

The quantification of tocopherol content was according to the Chinese Standard GB 5009.82 2016 [39]. Initially, approximately 0.8 g of the oil sample was placed into a 25 mL brown volumetric flask, followed by the sequential addition of 0.1 g of BHT and 10 mL of n-hexane. After vortexing to dissolve, the mixture was diluted to volume with n-hexane up to the mark on the volumetric flask. Finally, it was thoroughly vortexed and filtered using a 0.22 μm PTFE disposable syringe filter.

For the separation of tocopherols, an HPLC method employing a Zorbax Rx-SIL column (5 μm, 4.6 mm × 250 mm., Hewlett-Packard, Palo Alto, CA, USA) equipped with a 1260 Infinity II Fluorescence Detector (FLD, Agilent, Palo Alto, CA, USA) was used. A mixture of n-hexane and 1,4-dioxane (95/5, *v*/*v*) at a flow rate of 0.8 mL/min was employed as an isocratic mobile phase. The fluorescence excitation and emission wavelengths were 294 nm and 328 nm, respectively. The injection volume was 10 μL. Calibration curves were used for each component based on the concentrations of the analyzed samples as follows: α-tocopherol, Y = 57,370X − 9288.3 (R^2^ = 0.9998); β-tocopherol, Y = 82,173X − 3842.2 (R^2^ = 0.9998); γ-tocopherol, Y = 76,185X − 12,675 (R^2^ = 0.9999); δ-tocopherol, Y = 86,276X − 9753.5 (R^2^ = 0.9998).

#### 2.4.3. Squalene Analysis

Initially, 300 μL of a squalane internal standard solution (1 mg/mL in hexane) was added to a 250 mL tube and dried with nitrogen. Subsequently, 1.5 g of the sample oil and 50 mL of potassium hydroxide solution (1 mol/L in ethanol) were added to the tube, and the reaction was allowed to proceed for 50 min in an 80 °C water bath. Afterward, 50 mL of distilled water was added to terminate saponification. Hexane was employed for three washes, using 50 mL, 30 mL, and 20 mL, successively, after cooling to room temperature. The hexane extracts were amalgamated into a 250 mL separatory funnel, and 25 mL of ethanol was utilized for washing until the lower effluent reached a neutral state. Then, the washed hexane extracts were passed through an anhydrous sodium sulfate column to eliminate water and dried with nitrogen. Finally, hexane was used to dissolve the residue, and the volume was adjusted to 10 mL for GC analysis [40]. 

Squalene analysis was conducted using an Agilent 7890 Gas Chromatograph (Santa Clara, CA, USA) equipped with a hydrogen flame ionization detector (FID) and a HP-5 capillary column (30 mm × 0.32 mm, 0.25 μm; Agilent Technologies, Palo Alto, CA, USA). High-purity nitrogen served as the carrier gas, with a lateral pressure of 16 psi and a split ratio of 1:10. The injection and detector temperatures were set at 250 °C and 300 °C, respectively. The oven conditions were programmed as follows: the oven temperature increased from 160 °C to 220 °C at a rate of 15 °C/min and held for 2 min; then raised to 280 °C at a rate of 5 °C/min and held for 20 min; finally reaching 300 °C at a rate of 5 °C/min and held for 2 min. The content of squalene in the oil sample was calculated using the standard curve, wherein the peak area ratio of squalene to squalene served as the abscissa, and the mass ratio served as the ordinate (Y = 1.0257X − 0.0042; R^2^ = 0.9998).

#### 2.4.4. Polyphenol Analysis

Polyphenols in the oil samples were extracted following the standard of LS/T 6119-2017 [41]. Briefly, 2 g of oil sample was accurately weighed and dissolved in 6 mL of hexane. Subsequently, the solution was passed through a glycol-based solid-phase extraction column (500 mg, 3 mL; Waters Corporation, Milford, MA, USA) at a flow rate of 1 mL/min. Afterward, 10 mL of hexane was used to wash the extraction column, followed by the addition of 10 mL of a methanol solution for elution to collect the eluate. The eluate was dried using nitrogen, and the residue was dissolved in 2 mL of a methanol solution (50% in distilled water). The residue was then centrifuged at 10,000 rpm for 5 min at 4 °C after storage for 16 h at –18 °C, and then, the upper layer (methanol and water) was collected for analysis.

For the analysis of the polyphenol content [41,42], 1 mL of the extracted solution was transferred into a tube, followed by the addition of 0.5 mL of Folin–Ciocalteu reagent, 2 mL of 7.5% sodium carbonate solution, and 6.5 mL of distilled water in sequence. The mixture was vortexed for 1 min, and the tube was incubated for 30 min in a 70 °C water bath. After incubation, the absorbance was measured at 750 nm. The content of total phenolics was expressed as milligrams of a gallic acid equivalent per kilogram of oil sample (mg GAE/kg).

### 2.5. Volatile Compound Analysis

Headspace solid-phase microextraction (HS-SPME) was employed to extract volatile compounds from the oil samples [31,43] with slight modifications. Initially, 5 g of the oil sample was precisely weighed into a 5 mL headspace vial, together with 30 mg of 2-methyl-3-heptanone (20 mg/mL) serving as an internal standard. The vial was subsequently sealed and equilibrated for 10 min at 60 °C. Following this, volatile aroma compounds were extracted using SPME with a 50/30 µm divinylbenzene/carboxen/polydimethylsiloxane (DVB/CAR/PDMS) fiber (Supelco, Inc., Bellefonte, PA, USA) for 30 min at 60 °C.

To analyze the volatile compounds in the oil samples, a GC-MS system (7890B-5977B, Agilent, Santa Clara, CA, USA) equipped with a Stabilwax column (60 m × 0.25 mm × 0.25 µm, Restek Corp. Bellefonte, PA, USA) was employed. The fiber was desorbed into the GC port for 25 min at 250 °C before analysis using GC-MS in spitless injection mode. High-purity helium served as the carrier gas at a flow rate of 1.2 mL/min. The oven conditions were programmed as follows: the oven temperature increased from 40 °C to 240 °C at a rate of 5 °C/min and was held for 15 min. The transfer line and ion source temperatures were set at 245 °C and 250 °C, respectively. A full-scan mode of 33–350 *m*/*z* was performed to acquire the data, and the data were characterized using a combination of the relative retention index (RI value) and mass spectrum matching according to the NIST 2017 mass spectral library.

### 2.6. Antioxidant Activity Analysis 

#### 2.6.1. DPPH (1,1-diphenyl-2-picrylhydrazyl) Radical Scavenging Assay

The DPPH radical scavenging ability was determined following the instructions provided with the assay kit. Briefly, 0.4 mL of the oil sample and 0.6 mL of DPPH radical solution were combined in a tube and thoroughly mixed. After storing in the dark at room temperature for 30 min, the mixture was centrifuged at 12,000 rpm for 5 min at room temperature. Subsequently, 800 μL of the supernatant was transferred into a glass cuvette, and the absorbance value was measured at 517 nm, with 80% methanol serving as a control. The standard curve was constructed using concentrations of 0, 5, 10, 15, 20, and 25 μg/mL of Trolox (Y = 1.2639X − 0.7915; R^2^ = 0.9895). The DPPH radical scavenging abilities of the oil samples were calculated using the following formulas:

(1) $$DPPH\;radical\;scavenging\;ability\left(\%\right)=[1-(A_{1}-A_{2})/A_{3}]\times 100$$

(2) $$DPPH\;radical\;scavenging\;ability\left(\mu g\;Trolox/mL\right)=(Y+0.7915)/1.2639\times A_{4}$$

In the formulas, A_1_, A_2_, and A_3_ represent the absorbance values of the oil sample, control, and blank, respectively; Y denotes the DPPH radical scavenging ability (%); A_4_ signifies the dilution factor.

#### 2.6.2. ABTS (3-ethylbenzothiazoline-6-sulfonicacid) Radical Scavenging Assay

The ABTS radical scavenging ability was evaluated using an assay kit. To prepare the ABTS working solution, 5 mL of ABTS solution (7 mmol/L) and 88 μL of potassium persulfate solution (140 mmol/L) were thoroughly mixed and incubated for 12 h in darkness at 4 °C. Before use, the working solution was diluted with ethanol to achieve an absorbance value of 0.70 ± 0.05 nm at 734 nm for the ABTS working solution (30 °C). Subsequently, 50 μL of the oil sample was combined with 950 μL of the ABTS working solution and allowed to react in darkness for 6 min at room temperature. The absorbance was then measured at 734 nm, with an ethanol solution used as a control. A standard curve (Y = 0.4365X + 1.2067; R^2^ = 0.9973) was prepared using the different concentrations of Trolox standard solutions (0, 20, 40, 60, 80, 100 μg/mL). The ABTS radical scavenging abilities of the oil samples were calculated using the following formulas:

(3) $$ABTS\;radical\;scavenging\;ability\left(\%\right)=[1-(A_{1}-A_{2})/A_{3}]\times 100$$

(4) $$ABTS\;radical\;scavenging\;ability\left(\mu g\;Trolox/mL\right)=(Y–1.2067)/0.4365\times A_{4}$$

In the formulas, A_1_, A_2_, and A_3_ represent the absorbance values of the oil sample, control, and blank, respectively; Y denotes the ABTS radical scavenging ability (%); A_4_ signifies the dilution factor.

#### 2.6.3. Ferric Reducing Antioxidant Power (FRAP) Assay

The FRAP assay was conducted following the procedure outlined in the assay kit. Briefly, 75 μL of the oil sample, 75 μL of distilled water, and 850 μL of chromogenic solution were sequentially added to the tube and thoroughly mixed. Subsequently, the tube was allowed to react for 10 min at room temperature, and the absorbance was measured at 590 nm. The blank consisted of 150 μL of distilled water and 850 μL of the chromogenic solution. A standard curve (Y = 0.0284X − 0.0005; R^2^ = 0.9977) was established using the different concentrations of Trolox standard solutions (0, 15, 30, 45, 60, 75 nmol). The FRAP values of the oil samples were calculated using the following formulas:

(5) $$Sample\;absorbance=A_{1}-A_{3}$$

(6) $$FRAP\left(\mu mol\;Trolox/mL\right)=\frac{Y+0.0005}{0.0284\times V_{1}}\times 0.001\times A_{4}$$

In the formulas, A_1_ and A_3_ represent the absorbance values of the oil sample and blank, respectively; Y denotes the sample absorbance; V_1_ represents the volume of the oil sample; A_4_ signifies the dilution factor.

### 2.7. Statistical Analysis

All results were presented as the mean ± standard deviation (SD). Differences were analyzed using a one-way analysis of variance (ANOVA), followed by Duncan’s multiple range test, with significance set at *p* < 0.05. Correlations were assessed using Pearson’s rank correlation coefficient (*r*), and differences were further analyzed using principal component analysis (PCA). Data analysis was conducted using the Statistical Package for the Social Sciences (Version 27, SPSS Inc., Chicago, IL, USA), as well as the R packages ggbiplot, corrplot, and gplots (Version 4.4.1, R Foundation for Statistical Computing, Vienna, Austria).

## 3. Results and Discussion

### 3.1. Comparison of Glyceride Distributions

**Figure 1:**
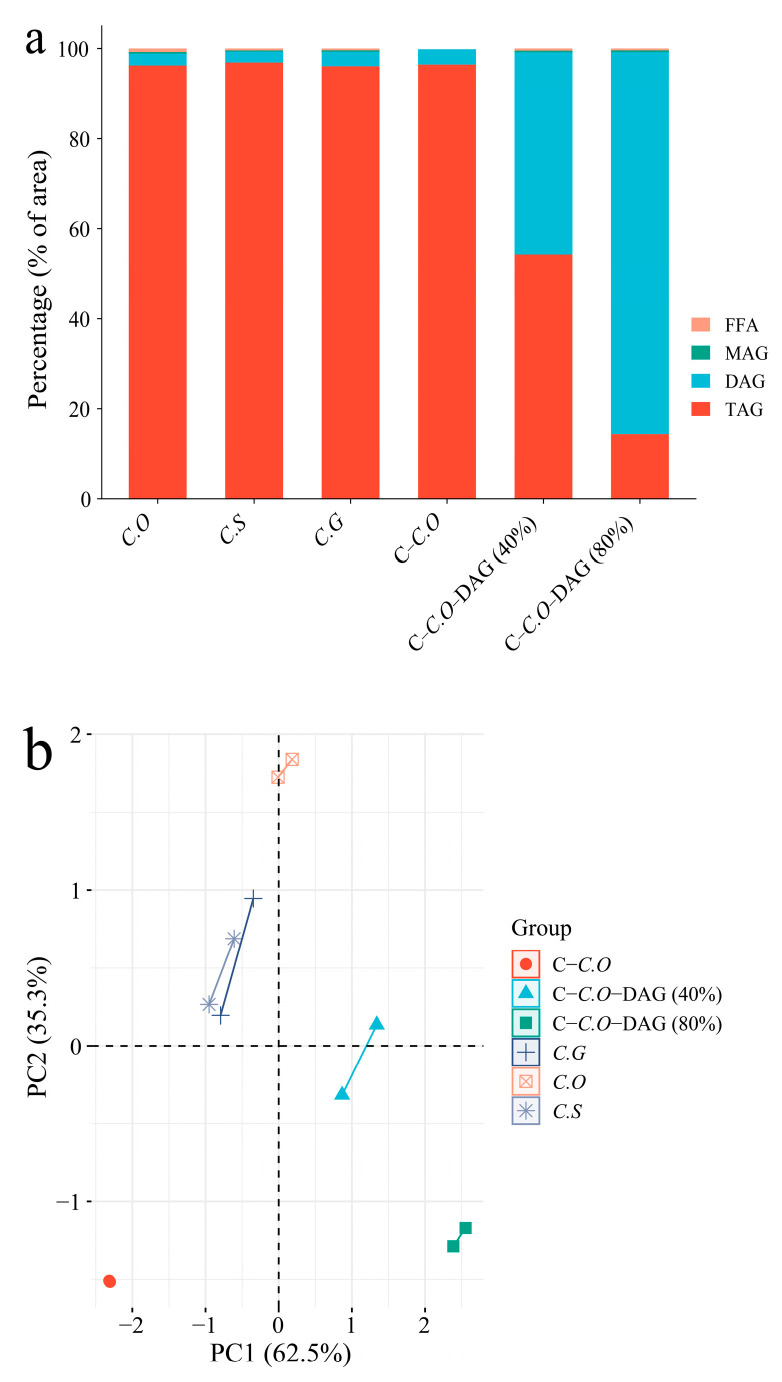
Comparison of glyceride distributions. (**a**) Glyceride distributions of different types of camellia oils, and (**b**) principal component analysis (PCA). The composition of TAG, DAG, MAG, and FFA of each type of camellia oils were used as variables. PC1 and PC2 factors (the first and second principal components, respectively) refer to the ordering scores obtained from the samples. PC1 accounts for 62.5% and PC2 for a further 35.3%. C.O, *C. oleifera*; C.S, *C. semiserrata*, C.G, *C. gauchowensis*, C-C.O, commercial *C. oleifera*; C-C.O-DAG (40%), commercial *C. oleifera* diacylglycerol oil (40%), and C-C.O-DAG (80%), commercial *C. oleifera* diacylglycerol oil (80%).

The glyceride distributions of each type of camellia oil are illustrated in Figure 1, indicating a high composition (96.04–96.88%) of TAG and a small amount of DAG (2.63–3.43%) in C.O, C.S, C.G, and C-C.O. This corresponds to a previous study demonstrating that DAG is a natural component of glycerides in various fats and oils, typically present at levels less than 10% [44]. Additionally, it was anticipated that no monoacylglycerols (MAGs) and free fatty acids (FFAs) would be detected in C-C.O, as commercial oil usually undergoes refining and other processes to remove phospholipids, FFA, and pigments. Regarding DAG-enriched camellia oils, the DAG composition in C-C.O-DAG (80%) was found to be up to 84.83%, with a small amount of MAG and FFA. C-C.O-DAG (40%), obtained from the mixture of C-C.O and C-C.O-DAG (80%), exhibited 44.89% of DAG (Figure 1a). 

Furthermore, by utilizing the composition of TAG, DAG, MAG, and FFA as variables for principal component analysis (PCA) (Figure 1b), it can be clearly distinguished that the three varieties of camellia oil (C.O, C.S, and C.G) differ from commercial refined camellia oil (C-C.O) and DAG-enriched camellia oils [C-C.O-DAG (40% and 80%)]. This suggested that the glyceride distributions can serve as useful markers for distinguishing between different types of camellia oils.

### 3.2. Comparison of TAG Profiles 

The relative abundances of TAGs in different types of camellia oils, including *C. oleifera* (C.O), *C. semiserrata* (C.S), *C. gauchowensis* (C.G), and commercially refined *C. oleifera* (C-C.O), were analyzed to compare the differences among the varieties, as summarized in Table 1. The dominant TAG across all camellia oils was OOO, constituting 43.42% to 49.14% of the total TAG content, representing nearly half of the total TAG levels. This is consistent with the range reported in the literature (28.50–69.80%) [16,17,45]. Additionally, TAGs in all camellia oils were primarily composed of UUU and SUU, with relatively lower levels of SUS and SSS. These findings aligned with a previous study [17], where the sum of UUU and SUU accounted for 91.32% of the total TAGs, significantly higher than the sum of the SUS and SSS content (8.76%) (*p* < 0.05), suggesting that TAGs in vegetable oils are primarily composed of UUU and SUU, while the levels of SUS and SSS are comparatively lower [16,17,45].

In addition, the distinct differences in TAG profiles were observed. In the LLL category, C.O exhibited the highest relative abundance of 0.9%, while C.S had the lowest at 0.57%. Similarly, in the OLL and PLL categories, C.O showed higher abundances compared to other varieties. Conversely, C.S displayed higher levels of OOL and SOO/SSL categories, suggesting that C.S may be a suitable choice for individuals seeking oils with a high monounsaturated fatty acid content, such as oleic acid. Of particular interest, C.G demonstrated a distinct TAG profile, with the highest levels of PPL, POL, PPL, POO, PPO, and POS categories compared to other varieties. The higher levels of saturated and unsaturated fatty acids, such as palmitic (P) and oleic (O) acids, in specific TAG species, like PPL and POL, may contribute to a more stable oil composition. This stability could be advantageous for dietary applications that require specific fatty acid profiles. Additionally, these TAG species are commonly linked to skin-conditioning properties, which position C.G. oil as a promising candidate for cosmetic formulations [46]. Notably, C-C.O displayed the highest abundance of OOO at 49.14%. However, it showed intermediate values across most categories, except for OOO, indicating a balanced TAG profile. The differences in TAG abundances among the varieties can be attributed to genetic factors, environmental conditions, and processing methods [20]. For example, the higher abundance of OOO in C-C.O compared to other varieties may be due to either processing methods or specific genetic traits that influence the synthesis of oleic acid [20,47].

**Figure 2:**
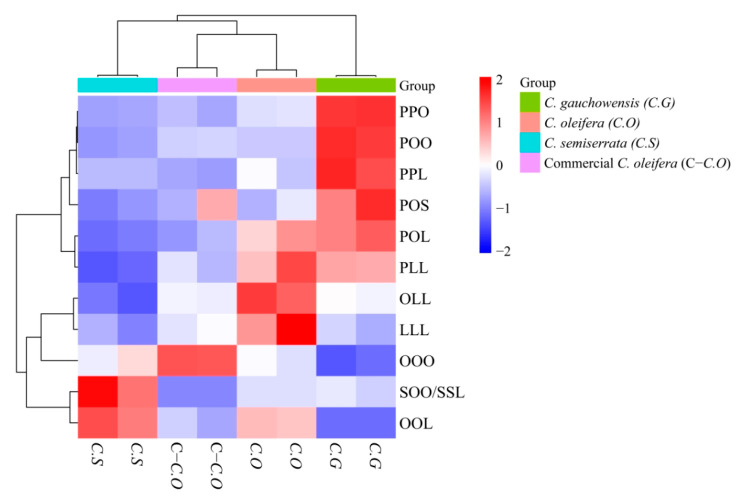
Cluster heat map of triacylglycerol profile of different types of camellia oils.

The cluster analysis, which employed dimensionality reduction using the TAG profiles, was undertaken to address the shielding effect resulting from the absolute dominance of major TAGs. The results are illustrated in Figure 2, revealing distinct characteristics for each camellia oil variety. C.O was notably enriched in PLL, OLL, and LLL. In contrast, C.G exhibited a preference for PPO, POO, and PPL, suggesting a different fatty acid composition compared to C.O. Furthermore, the major TAG in C-C.O was OOO, while C.S showed higher levels of OOL and SOO/SSL. These findings suggested that the TAG profiles of each camellia oil variety can serve as unique fingerprints for differentiating and evaluating the characteristics of different types of camellia oils. Similarly, Wei et al. [17] showed that a distinct pattern of TAG spectra for vegetable oils was obtained, allowing for the classification of 18 edible vegetable oils into five groups based on their TAG profiles.

### 3.3. Comparison of Bioactive Components

#### 3.3.1. Fatty Acids

The fatty acid composition of oils plays a crucial role in determining their nutritional values and functional properties. Table 2 presents the distributions of fatty acids on the glycerol backbone of different types of camellia oils. Across all types of camellia oils, the predominant fatty acids were oleic acid (C18:1 n-9), linoleic acid (C18:2 n-6), and palmitic acid (C16:0). These findings aligned with previous reviews on the fatty acid composition of camellia oils, which consistently highlight oleic acid (C18:1 n-9), linoleic acid (C18:2 n-6), and palmitic acid (C16:0) as the main fatty acids in these oils [2,20]. This also corresponded to the TAG profiles found in camellia oil, which were predominantly composed of these three fatty acids linked to the glycerol backbone (Table 1).

Among the varieties of total fatty acids, C.S exhibited the highest levels of monounsaturated fatty acids (MUFAs), primarily due to its elevated oleic acid content. In contrast, C.G had the highest levels of saturated fatty acids (SFAs), mainly attributed to its high palmitic acid content. C.O showed intermediate levels of both MUFAs and SFAs. These variations in fatty acid composition among the camellia oil varieties could impact their nutritional profiles and suitability for different culinary applications. The high oleic acid content in camellia oils, particularly in C.S, makes them a healthy choice, as oleic acid is a MUFA associated with various health benefits, including a reduced risk of cardiovascular diseases [3]. Additionally, the relatively low levels of saturated fatty acids (SFAs) in camellia oils, compared to other edible oils, like palm oil or coconut oil, make them a healthier alternative for consumption [17]. Comparing the commercially refined *C. oleifera* oil (C-C.O) with its DAG-enriched counterparts, it was evident that the enrichment process significantly alters the fatty acid composition, particularly decreasing the relative abundance of SFAs and increasing the relative abundance of unsaturated fatty acids (UFAs) (Table 2). The results were in line with previous studies showing that DAG-enriched oils had a higher content of UFAs, which may contribute to their potential health benefits, such as improved lipid metabolism [33,34].

In addition, a distinct distribution of fatty acids at the sn-2 position was observed among the varieties. For example, C.O exhibited the highest content of sn-2 palmitic acid (C16:0) among the three varieties, resulting in the highest level of SFAs at the sn-2 position. In contrast, C.S had the highest level of MUFAs at the sn-2 position, mainly due to its high oleic acid content. Furthermore, C.G contained a higher level of sn-2 linoleic acid (14.90%) compared to that in C.O (12.95%) and C.S (11.25%), resulting in the highest PUFA level at the sn-2 position observed in C.G (Table 2). The differences in fatty acid distribution among camellia oil varieties, especially in the sn-2 position, are noteworthy. The sn-2 position is known to affect the bioavailability and metabolism of fatty acids [48]. Therefore, these differences could influence the digestion and absorption kinetics of these oils. 

Considering the fatty acid distributions of each camellia oil on the glycerol backbones, it had been documented that UFAs preferred to be attached at the sn-2 position, while SFAs were concentrated at the sn-1,3 position in vegetable oils, suggesting that the fatty acid distributions on the glycerol backbones were not arbitrary, as indicated by Wei et al. [17], highlighting a structured and potentially purpose-driven arrangement of fatty acids in TAGs. The findings were consistent with the results of this study, as shown in Table 2, as the compositions of SFAs, MUFAs, and PUFAs at the sn-2 position among C.O, C.S, C.G, and C-C.O showed a range of 1.66–5.61%, 81.15–86.85%, and 11.43–15.23%, respectively, which were higher than those in the sn-1,3 position (15.16–20.10%, 72.63–76.03%, and 7.29–9.21%, respectively). When considering DAG-enriched camellia oils, commercial *C. oleifera*-DAG (40% and 80%), it had been reported that 1,2-(or 2,3)-diacyl-sn-glycerol (1,2-DAG) and 1,3-diacyl-sn-glycerol (1,3-DAG) typically maintain a dynamic equilibrium through acyl migration during enzymatic processes and in the presence of acid, alkali, or heat, with a ratio of 1,2-DAG to 1,3-DAG of approximately 7:3 [49]. Therefore, differences in the distribution of fatty acids on the glycerol backbone were expected in DAG-enriched camellia oils (Table 2).

**Figure 3:**
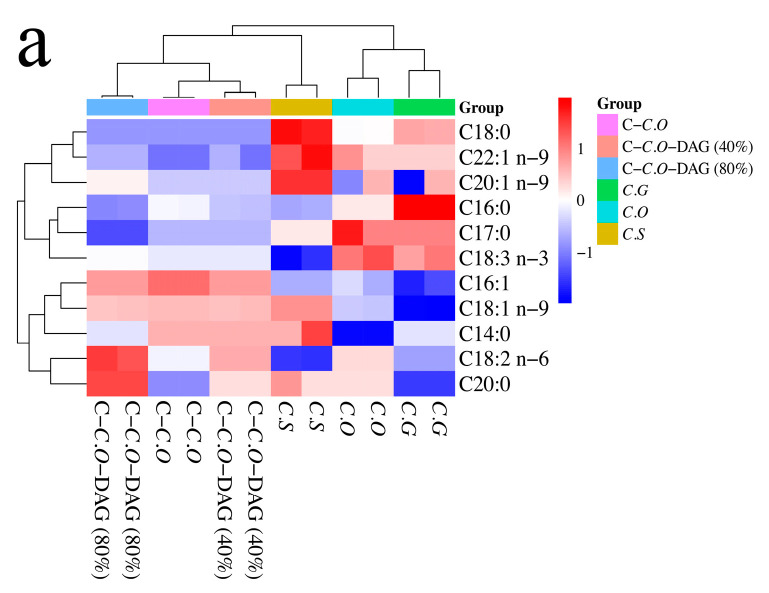

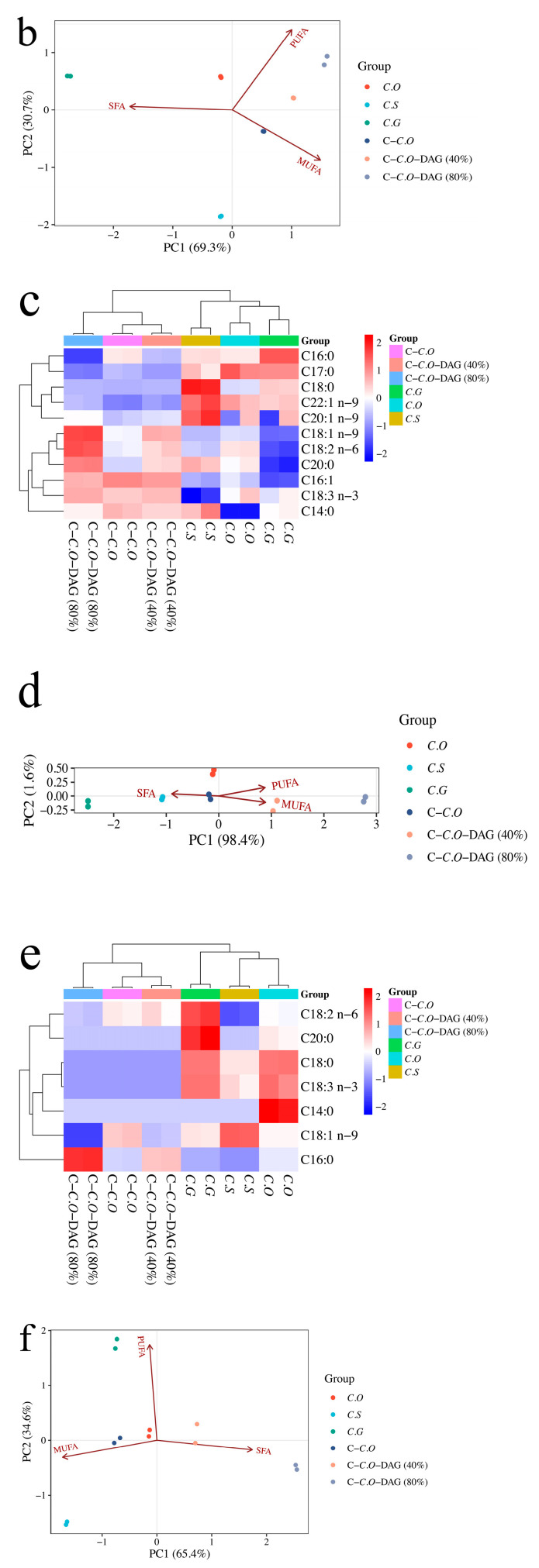
Cluster heat map and principal component analysis (PCA) were conducted for six types of camellia oils using the following variables: (**a**) cluster heat map of the total fatty acids, (**b**) PCA of total SFAs, MUFAs, and PUFAs, (**c**) cluster heat map of the sn-1,3 position of fatty acids, (**d**) PCA of sn-1,3 position of total SFAs, MUFAs, and PUFAs, (**e**) cluster heat map of the sn-2 position of fatty acids, and (**f**) PCA of the sn-2 position of fatty acids of total SFAs, MUFAs, and PUFAs. SFAs, saturated fatty acids; MUFAs, monounsaturated fatty acids; PUFAs, polyunsaturated fatty acids; C.O, *C. oleifera*; C.S, *C. semiserrata*), C.G, *C. gauchowensis*; C-C.O, commercial *C. oleifera*; C-C.O-DAG (40%), commercial *C. oleifera* diacylglycerol oil (40%); and C-C.O-DAG (80%), commercial *C. oleifera* diacylglycerol oil (80%).

The distribution characteristics of fatty acids in different varieties of camellia oils and DAG-enriched camellia oils were analyzed using cluster heat map and principal component analysis (PCA) with the distributions of fatty acids on different glycerol backbones as variables, as shown in Figure 3. The results revealed distinct groupings among the six types of oils based on their total fatty acid composition. Firstly, C.O and C.G form one group, characterized by relatively high levels of palmitic acid (C16:0), α-linolenic acid (C18:3 n-3), and margaric acid (C17:0). Secondly, C-C.O and its DAG-enriched counterparts (C-C.O-DAG 40% and 80%) form another group with relatively high levels of palmitoleic acid (C16:1) and linoleic acid (C18:2 n-6). Lastly, represented by C.S, it exhibited higher levels of n-9 fatty acids (C18:1 n-9, C20:1 n-9, and C22:1 n-9) and SFAs (C14:0, C18:0, and C20:0) (Figure 3a). Accordingly, the results of PCA using total SFAs, MUFAs, and PUFAs as variables showed that C.O, C.S, and C.G were characterized by high levels of SFA among different types of camellia oils. In contrast, total MUFAs and PUFAs were representative of C-C.O and its DAG-enriched counterparts, especially C-C.O-DAG 40% and 80% (Figure 3b), suggesting that the relative content and saturation of fatty acids in camellia oil can be used as indicators to distinguish different types of camellia oil [20,45]. For instance, Shi et al. [20] demonstrated that the content of oleic acid (C18:1 n-9) in *C. oleifera* (77.12–79.50%) and *C. chekiangoleosa* (75.97–79.33%) was significantly higher compared to *C. sinensis* (49.07–56.27%) (*p* < 0.05). Conversely, the content of palmitic acid (C16:0) in *C. oleifera* (8.37–8.92%) and *C. chekiangoleosa* (7.45–9.06%) was notably lower than in *C. sinensis* (13.52–17.55%).

Furthermore, when considering the distribution of specific fatty acids at the sn-1,3 position, C-C.O and its DAG-enriched counterparts (C-C.O-DAG 40% and 80%) could be clearly differentiated based on their fatty acid profiles. For instance, C-C.O and C-C.O-DAG (40%) were distinguished by their high levels of palmitoleic acid (C16:1) and α-linolenic acid (C18:3 n-3), respectively, while C-C.O-DAG (80%) exhibited notably high levels of linoleic acid (C18:2 n-6) and oleic acid (C18:1 n-9) (Figure 3c), similar to total fatty acids. In addition, PCA of the total SFAs, UFAs, and PUAs at the sn-1,3 positions clearly showed that the DAG-enriched counterparts (C-C.O-DAG 40% and 80%) had a significant composition of unsaturated fatty acids, including MUFAs and PUFAs (Figure 3d).

In contrast, the three varieties of camellia oils exhibited distinct fatty acid profiles at the sn-2 position. As depicted in Figure 3e,f, C.S showed a preference for oleic acid (C18:1 n-9), C.O was characterized by higher levels of C14:0, and C.G contained elevated levels of C20:0 and linoleic acid (C18:2 n-6). Moreover, C-C.O and its DAG-enriched counterparts (40% and 80%) could be clearly differentiated based on the presence of oleic acid (C18:1 n-9), linoleic acid (C18:2 n-6), and palmitic acid (C16:0) at the sn-2 position, respectively (Figure 3e). It is worth noting that the degree of saturation of fatty acids at the sn-2 position in the DAG-enriched counterparts exhibited an opposite trend compared to the sn-1,3 positions. SFAs were more likely to be distributed at the sn-2 position, whereas the sn-1,3 positions were rich in unsaturated fatty acids (Figure 3d,f). The DAG-enriched counterparts were synthesized via enzymatic esterification using C-C.O as the raw material. Due to the low stability of unsaturated fatty acids [50], those formed via the hydrolysis of C-C.O may be more easily linked to the sn-1,3 positions on the glycerol backbone than SFAs under the catalysis of lipase, resulting in an increase in the content of unsaturated fatty acids and a decrease in SFAs at the sn-1,3 positions of the DAG-enriched counterparts. Therefore, according to the above findings, it was apparent that the distinctive distribution patterns of fatty acids on these glycerol backbones can be utilized as a criterion to differentiate between various types of camellia oils.

#### 3.3.2. Tocopherols, Squalene, and Polyphenols

**Figure 4:**
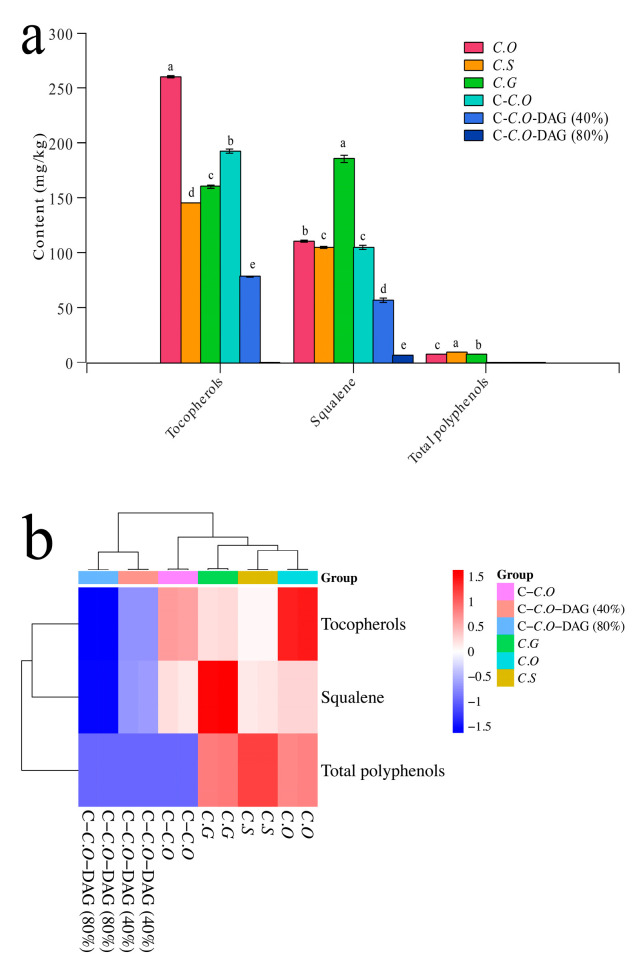
Comparison of tocopherols, squalene, and total polyphenols in different types of camellia oil (**a**), and cluster heat map with tocopherols, squalene, and total polyphenols in different types of camellia oil as variables (**b**). Mean values with different letters in are significantly different from each other at *p* < 0.05. C.O, *C. oleifera*; C.S, *C. semiserrata)*; C.G, *C. gauchowensis*; C-C.O, commercial *C. oleifera*; C-C.O-DAG (40%), commercial *C. oleifera* diacylglycerol oil (40%); and C-C.O-DAG (80%), commercial *C. oleifera* diacylglycerol oil (80%).

The contents of tocopherols, squalene, and total polyphenols among different types of camellia oils were compared, as depicted in Figure 4a. Tocopherols, renowned for their antioxidant properties, can be categorized into four forms, including α-, β-, γ-, and δ-tocopherol [3]. Interestingly, only α-tocopherol was identified in the six types of camellia oils examined in the present study, which is consistent with the findings of Tang et al. [31], who also exclusively detected α-tocopherol in seven varieties of camellia oils, with concentrations ranging from 112.8 to 257.8 mg/kg. The highest content of tocopherols was observed in C.O (260.50 mg/kg), followed by C-C.O (192.50 mg/kg) and C.G (160.00 mg/kg), with a significant difference (*p* < 0.05) noted between them. It is noteworthy that no tocopherols were detected in C-C.O-DAG (80%) compared with C-C.O, implying that tocopherols may degrade due to the high temperature of molecular distillation during the preparation of DAG-enriched camellia oil from C-C.O [16]. Indeed, Kmiecik et al. [51] have demonstrated that heating led to a reduction in tocopherol levels, with tocopherol changes dependent on the type of oil and the duration of heating. Additionally, a small amount of tocopherols was detected in C-C.O-DAG (40%), probably because C-C.O-DAG (40%) was obtained by mixing C-C.O and C-C.O-DAG (80%) in a specific ratio.

A study demonstrated that camellia oil (138.28 mg/kg) has a comparatively high squalene content compared to other edible oils, such as soybean oil (80 mg/kg) and rapeseed oil (30 mg/kg) [3]. In this study, the squalene content in C.O, C.G, C.S, and C-C.O ranged from 104.50 mg/kg to 185.50 mg/kg, aligning with earlier findings reporting squalene levels in camellia oil between 110 and 295 mg/kg [20]. Notably, C.G exhibited the highest squalene content (185.50 mg/kg), significantly surpassing other camellia oil types (C.O, C.S, and C-C.O; 104.50–110.50 mg/kg) (Figure 4a), highlighting the influence of camellia oil variety on the squalene content. Moreover, DAG-enriched camellia oils (C-C.O-DAG) showed very low squalene contents, indicating an impact from both the variety and processing techniques on squalene levels.

Polyphenols are known for their health benefits, including antioxidant properties [10]. According to their chemical structure, polyphenols can be categorized into catechins, flavonoids, anthocyanins, and phenolic acids [2]. To date, 24 phenolic compounds have been identified from camellia oil, including 13 phenolic acids (i.e., benzoic acid, p-hydroxybenzoic acid, protocatechuic acid, gallic acid, phthalic acid, p-hydroxyphenylacetic acid, vanillic acid, cinnamic acid, p-coumaric acid, caffeic acid, ferulic acid, sinapic acid, and chlorogenic acid), 4 catechins (i.e., catechin, epicatechin, epigallocatechin, and epigallocatechin gallate), and 7 flavonoids (i.e., apigenin, kaempferol, luteolin, quercetin, myricetin, naringenin, and taxifolin) [20,52]. Notably, benzoic acid (2.95–18.87 mg/kg), vanillic acid (1.63–17.64 mg/kg), cinnamic acid (3.72–16.69 mg/kg), and naringenin (0.16–10.77 mg/kg) collectively contribute to more than half of the total polyphenol content, highlighting their contribution to the nutritional value and shelf life of camellia oil [20,52]. In this study, various distributions of total polyphenols in different types of camellia oils were observed, as illustrated in Figure 4a. Total polyphenols were detected in C.O, C.G, and C.S, with the highest amount found in C.S, followed by C.G and C.O. Notably, no polyphenols were observed in C-C.O, suggested that the refining process, particularly the temperatures used, may play a significant role in the presence or absence of polyphenols in camellia oil. As polyphenols are highly unstable and can be easily degraded during post-harvesting and processing, it is crucial to consider the impact of refining methods on their preservation. Correspondingly, Zeng et al. [16] reported that the total polyphenol content in refined camellia oil (not detected) was significantly reduced compared with cold-pressed camellia oil (19.15 mg/kg). Additionally, it was expected that no polyphenols would be detected in C-C.O-DAG due to the absence of polyphenols in C-C.O (Figure 4a). As demonstrated above, various camellia oils are rich in bioactive components, such as tocopherols, squalene, and polyphenols. Therefore, for those looking to increase their antioxidant intake, using these camellia oils is recommended. On the other hand, although DAG-enriched oils contain reduced levels of bioactive components, like tocopherols, squalene, and polyphenols, DAG-enriched oils have been proven beneficial for fat metabolism and blood lipid control [34]. Additionally, this study shows that DAG-enriched oils contain higher levels of unsaturated fatty acids (Table 2), making them a good choice for individuals with chronic conditions, such as cardiovascular diseases [34].

Cluster analysis, utilizing tocopherol, squalene, and total polyphenol contents as variables, revealed distinct distributions among different camellia oils (Figure 4b). Specifically, C.S was characterized by its high total polyphenol content, C.G by its squalene content, and C.O by its tocopherol content. In contrast, low contents of tocopherol, squalene, and total polyphenols were characteristic of C-C.O and DAG-enriched counterparts, in the order of C-C.O-DAG 80% < C-C.O-DAG 40% < C-C.O. These findings suggested that tocopherol, squalene, and total polyphenols are useful indicators for distinguishing different types of camellia oils.

#### 3.3.3. Cluster Analysis of Bioactive Components 

**Figure 5:**
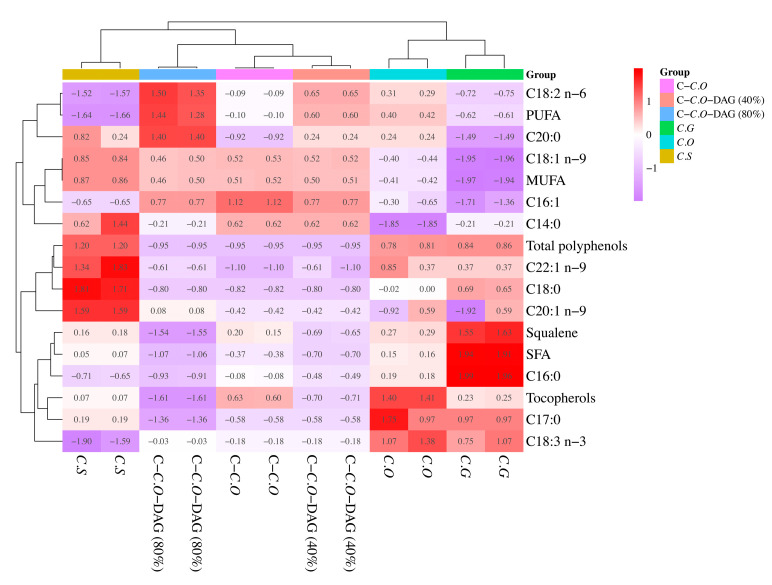
Cluster heat map of bioactive components of different types of camellia oils. C.O, *C. oleifera*; C.S, *C. semiserrata*); C.G, *C. gauchowensis*; C-C.O, commercial *C. oleifera*; C-C.O-DAG (40%), commercial *C. oleifera* diacylglycerol oil (40%); and C-C.O-DAG (80%), commercial *C. oleifera* diacylglycerol oil (80%).

Since fatty acids, tocopherols, squalene, and total polyphenols can serve as useful indicators for distinguishing different types of camellia oils, the strengths of these indicators were explored using the bioactive components in different oil types as variables. As shown in Figure 5, the representative features of the bioactive components for different oils exhibited varying strengths. For instance, high levels of squalene, palmitic acid (C16:0), and SFAs can serve as indicators of C.G, in the order of palmitic acid (C16:0) > SFA > squalene. In contrast, the primary indicator of C.S was C18:0, followed by C22:1 n-9, C20:1 n-9, and total polyphenols. Conversely, linoleic acid (C18:2 n-6), PUFAs, and C20:0 were the indicators for C-C.O-DAG 80%, showing a similar order. The highest indicator of bioactive components for C.O and C-C.O was C16:1 and C17:0, respectively (Figure 5). Therefore, different types of camellia oils can be distinguished more accurately based on the strength of their representative bioactive components.

### 3.4. Comparison of Volatile Compounds

The volatile compounds, a key sensory characteristic that varies across different types of camellia oil, were detected and identified. The analysis results revealed the presence of 157 volatile compounds in various types of camellia oil, including C.O, C.S, C.G, C-C.O, C-C.O-DAG (40%), and C-C.O-DAG (80%) (Table S1). Among these, C.O primarily contained six volatile compounds, namely octane (1.85 mg/kg), nonanal (1.77 mg/kg), benzene acetaldehyde (1.23 mg/kg), 1-butanol (1.17 mg/kg), hexanal (1.11 mg/kg), and ethyl tiglate (1.06 mg/kg). Conversely, C.S was characterized by acetic acid (4.48 mg/kg), 2,3-butanediol (1.16 mg/kg), and hexanal (1.07 mg/kg) as its main components. Furthermore, ethanol (6.59 mg/kg), acetic acid (2.52 mg/kg), heptanal (1.41 mg/kg), 1-butanol (1.36 mg/kg), butanoic acid (1.29 mg/kg), D-limonene (1.11 mg/kg), and ethyl acetate (1.03 mg/kg) were identified as the major volatile compounds in C.G. 

In addition, different volatile composition profiles were observed between refined camellia oil (C-C.O) and cold-pressed camellia oils (C.O, C.S, and C.G), indicating that refining processes, such as degumming, neutralization, bleaching, and deodorization, substantially alter the odor profile of camellia oils [16]. Volatile compounds formed during refining are typically the result of thermal degradation and oxidation. For instance, alcohols, aldehydes, ketones, and other oxidation products are generated due to the exposure of camellia oil to high temperatures and the removal of natural antioxidants [53]. Specifically, volatile compounds, such as 1-penten-3-ol (0.04 mg/kg), 4-hydroxy-3-hexanone (0.02 mg/kg), 1,2-ethanediol (0.02 mg/kg), 1,4-butanediol (0.01 mg/kg), 3-heptanone (0.35 mg/kg), 1-pentanone (0.05 mg/kg), and 1-hydroxy-2-butanone (0.03 mg/kg), were found in refined camellia oil (C-C.O) but were not detected in cold-pressed camellia oils (C.O, C.S, and C.G) (Table S1). The refining process, particularly the deodorization step, can remove both useful and undesirable volatiles, resulting in a more neutral sensory profile compared to cold-pressed oils [16,53].

Notably, DAG-enriched camellia oils exhibited distinct volatile compound profiles, showcasing higher levels of ethanol (0.87–1.16 mg/kg) and octane (0.57–0.65 mg/kg), while having lower levels of 3-heptanone (not detected) and toluene (0.04–0.05 mg/kg) compared to C-C.O, which had levels of 0.11 mg/kg, 0.02 mg/kg, 0.35 mg/kg, and 0.37 mg/kg, respectively. These differences suggested that both the varieties of camellia oils and the technique of the DAG-enriched process significantly impact the volatile compounds of camellia oil [16,31].

**Figure 6:**
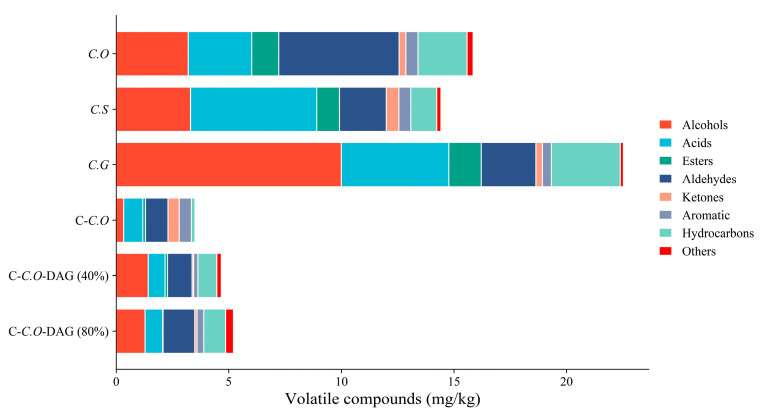
Classification of volatile compounds in different types of camellia oils. C.O, *C. oleifera*; C.S, *C. semiserrata*; C.G, *C. gauchowensis*; C-C.O, commercial *C. oleifera*; C-C.O-DAG (40%), commercial *C. oleifera* diacylglycerol oil (40%); and C-C.O-DAG (80%), commercial *C. oleifera* diacylglycerol oil (80%).

The volatile compounds present in different types of camellia oils, encompassing a range of organic compounds, including alcohols (25), acids (12), esters (16), aldehydes (16), ketones (14), aromatic (4), hydrocarbons (20), and other categories (5), are illustrated in Figure 6. Significant disparities in the volatile compound composition were observed among the different camellia oil samples. Notably, C.G exhibited markedly higher levels of alcohols (9.99 mg/kg) compared to C.O (3.20 mg/kg) and C.S (3.30 mg/kg), while C.S showcased elevated concentrations of acids (5.60 mg/kg), likely attributable to genetic variations and the growth environments among camellia oil varieties [54]. Furthermore, esters were found to have relatively low concentrations across most samples, with C.G displaying slightly higher levels. The presence of aromatics remained relatively consistent across C.O, C.S, and C.G, with minimal variation noted. Of particular interest was the substantially lower content of volatile compounds in C-C.O, indicative of the pronounced impact of the refining process on the volatile compound composition. Therefore, these findings suggested the potential utility of volatile compounds in distinguishing between different camellia oil varieties [31].

In addition, DAG-enriched camellia oils, specifically C-C.O-DAG (80%) and C-C.O-DAG (40%), exhibited significantly distinct volatile compound profiles compared to their raw oils (C-C.O). Notably, alcohols and hydrocarbons were more abundant in C-C.O-DAG (40% and 80%), whereas ketones and aromatics were present in lower amounts. These oils, derived from C-C.O through enzymatic hydrolysis and molecular distillation, possess a unique flavor profile attributed to the processing-induced alterations in the volatile compound composition. Enzymatic hydrolysis with lipase catalyzes the breakdown of triacylglycerol (TAG) into diacylglycerol (DAG), monoacylglycerol (MAG), and free fatty acids (FFAs) [36], resulting in the release of volatile compounds, including alcohols and hydrocarbons. Molecular distillation, conducted at high temperatures, further modifies the volatile profile by removing low-boiling-point components and inducing oxidative reactions [55]. For instance, the removal of some ketones and aromatics during this process resulted in their lower levels in DAG-enriched camellia oils. Additionally, a previous study has linked the presence of aldehydes, ketones, and heterocyclic compounds to the oxidation of unsaturated fatty acids, while alcohols are recognized as intermediate products of the Strecker degradation pathways [56]. Furthermore, high levels of unsaturated fatty acids (UFAs) across all the types of camellia oils were observed, as described in Table 2. Consequently, the distinctive flavor of DAG-enriched camellia oils can be attributed to the processing methods employed, highlighting the role of processing in shaping volatile compound profiles and flavor characteristics.

### 3.5. Comparison of Antioxidant Activity

**Figure 7:**
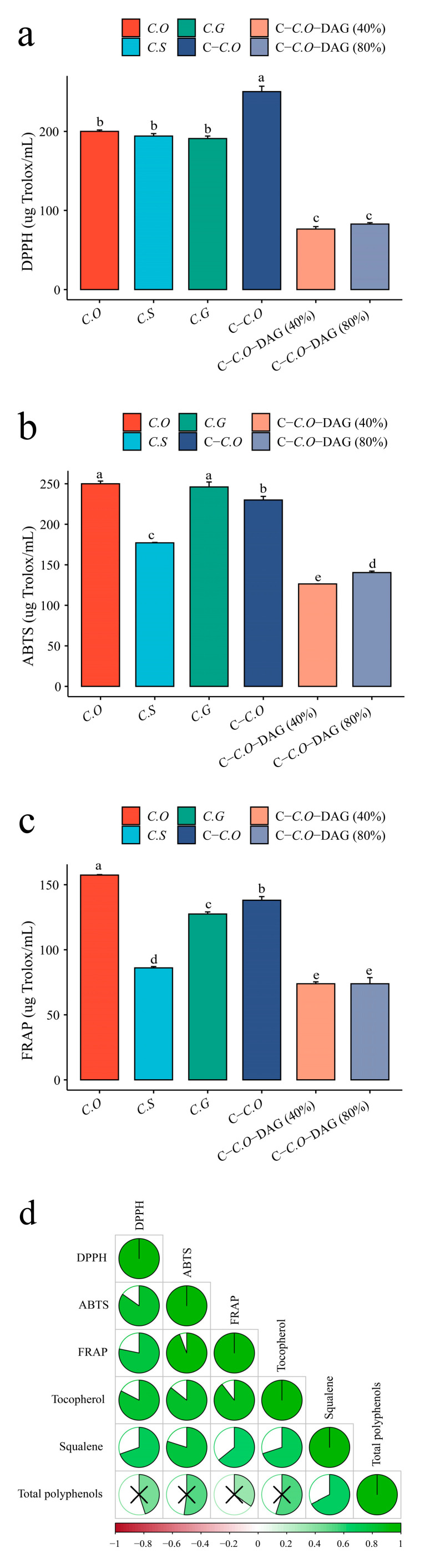
Antioxidant activity abilities of different types of camellia oils and correlated with their bioactive components. (**a**) DPPH radical scavenging abilities, (**b**) ABTS radical scavenging abilities, (**c**) FRAP abilities, and (**d**) correlations of between antioxidant activity abilities and bioactive components in different types of camellia oils. Mean values with different letters are significantly different from each other at *p* < 0.05. C.O, *C. oleifera*; C.S, *C. semiserrata*; C.G, *C. gauchowensis*; C-C.O, commercial *C. oleifera*; C-C.O-DAG (40%); commercial *C. oleifera* diacylglycerol oil (40%); and C-C.O-DAG (80%), commercial *C. oleifera* diacylglycerol oil (80%).

The antioxidant activities of various camellia oils were evaluated using DPPH, ABTS, and FRAP assays, as depicted in Figure 7a–c. In the DPPH assay, *C*-C.O exhibited the highest antioxidant activity, with a value of 250.30 μmol Trolox/mL (Figure 7a). Conversely, *C*. oleifera (C.O) demonstrated the highest activity in the ABTS and FRAP assays, with values of 249.99 μmol Trolox/mL and 157.41 μmol Trolox/mL, respectively (Figure 7b,c). These results underscore the significant influence of camellia oil varieties on their antioxidant capabilities [57], with C.O standing out as a notable source of bioactive compounds with high antioxidant potential. The presence of diacylglycerol (DAG) in DAG-enriched camellia oils (40% and 80%) derived from C-C.O resulted in notably lower antioxidant activities compared to other samples across all three assays (*p* < 0.05). This implies that the formation of DAG, occurring during the enzyme-catalyzed reaction and molecular distillation process, may diminish the overall antioxidant efficacy of the oils.

Camellia oil contains bioactive components, such as tocopherol, squalene, and polyphenols, known for their antioxidant effects [10,18,24,25,26]. The correlations between these components and antioxidant activity in various camellia oils were explored in this study (Figure 7d). As anticipated, strong positive correlations were observed, particularly between tocopherol and antioxidant activity, with coefficients of 0.90 (tocopherol and FRAP), > 0.86 (tocopherol and ABTS), and > 0.83 (tocopherol and DPPH) at *p* < 0.01. Furthermore, squalene exhibited a highly significant positive correlation with ABTS (*r* = 0.80; *p* < 0.01), potentially explaining the high ABTS radical scavenging ability of C.G (Figure 7b), as its squalene content was significantly higher than that of other types of camellia oils (p < 0.05) (Figure 4a). These results aligned with prior research highlighting the strong associations among vitamin E, squalene, and the antioxidant capacity of *Camellia* spp. [16,57]. For instance, Zeng et al. [16] reported a significant positive correlation of α-tocopherol with DPPH (polar extract: *r* = 0.977; non-polar extract: *r* = 0.754; and whole oil: *r* = 0.907), FRAP (*r* = 0.972), and ABTS (*r* = 0.821) at the 0.01 significance level. Interestingly, there were no significant correlations between total polyphenols and DPPH, ABTS, and FRAP (*p* > 0.05). The above findings suggested that the antioxidant activities of camellia oils are heavily influenced by their bioactive components, especially tocopherol and squalene, highlighting the potential health benefits associated with these oils [16,57]. Therefore, in the biosynthesis of DAG-enriched camellia oil, it is recommended to optimize process conditions, particularly the temperature, to maximize the retention of heat-sensitive tocopherols and polyphenols. This is crucial for enhancing the antioxidant activity of DAG-enriched oils and extending its shelf life.

## 4. Conclusions

This study shed light on the diverse characteristics of different varieties of camellia oils and their diacylglycerol (DAG)-enriched derivatives. Through a comprehensive analysis encompassing TAG species, bioactive components, volatile compounds, and antioxidant activity, notable distinctions were observed among six types of camellia oils, including three varieties of *C. oleifera* (C.O), *C. semiserrata* (C.S), and *C. gauchowensis* (C.G), along with commercially refined C. oleifera oil (C-C.O) and its DAG-enriched variants (at 40% and 80% enrichment). The results revealed unique patterns in TAG profiles, fatty acid distributions across different glycerol backbones, tocopherols, squalene, total polyphenols, and volatile compounds, suggesting their potential as discriminatory markers. Notably, DAG-enriched oils exhibited elevated levels of unsaturated fatty acids (UFA) while experiencing reductions in tocopherol, squalene, and total polyphenol contents compared to C-C.O. Furthermore, the enrichment process significantly influenced the composition of volatile compounds, with DAG-enriched oils (at 40% and 80% enrichment) demonstrating higher contents of alcohols and hydrocarbons, whereas they had lower contents of ketones and aromatics compared to their raw oils. Importantly, the study also elucidated the impact of DAG enrichment on antioxidant activity, with DAG-enriched oils displaying diminished abilities compared to their counterparts. Among the oils analyzed, C.O exhibited the highest antioxidant activity, followed by C.G, with strong correlations observed between antioxidant activity and tocopherol, as well as squalene content. Therefore, these findings contribute to a deeper understanding of the nutritional and functional properties of camellia oils and their DAG-enriched derivatives, providing valuable insights for their potential applications in the food, cosmetic, and pharmaceutical industries.

## Figures and Tables

**Table 1:** Relative abundances of TAGs (% of total TAGs) in different varieties of camellia oils.

TAG ^1^	ECN ^2^	*C. oleifera*	*C. semiserrata*	*C. gauchowensis*	*Commercial C. oleifera*
LLL	42	0.96 ± 0.14 ^a^	0.57 ± 0.04 ^b^	0.62 ± 0.04 ^b^	0.69 ± 0.03 ^b^
OLL	44	3.06 ± 0.04 ^a^	2.51 ± 0.03 ^c^	2.76 ± 0.02 ^b^	2.74 ± 0.00 ^b^
PLL	44	1.34 ± 0.13 ^a^	0.89 ± 0.02 ^b^	1.28 ± 0.01 ^a^	1.07 ± 0.05 ^b^
OOL	46	11.99 ± 0.03 ^b^	12.77 ± 0.28 ^a^	10.14 ± 0.02 ^d^	10.80 ± 0.25 ^c^
POL	46	4.80 ± 0.15 ^a^	4.11 ± 0.03 ^b^	5.01 ± 0.09 ^a^	4.27 ± 0.08 ^b^
PPL	46	0.57 ± 0.05 ^b^	0.52 ± 0.00 ^b^	0.90 ± 0.04 ^a^	0.48 ± 0.01 ^b^
OOO	48	45.83 ± 0.39 ^b^	46.33 ± 0.71 ^b^	43.42 ± 0.21 ^c^	49.14 ± 0.05 ^a^
POO	48	15.33 ± 0.01 ^b^	14.71 ± 0.10 ^c^	18.59 ± 0.17 ^a^	15.40 ± 0.07 ^b^
PPO	48	1.35 ± 0.02 ^b^	1.17 ± 0.00 ^c^	2.02 ± 0.01 ^a^	1.22 ± 0.05 ^c^
SOO/SSL	50	11.40 ± 0.00 ^b^	13.19 ± 0.56 ^a^	11.39 ± 0.15 ^b^	10.70 ± 0.01 ^b^
POS	50	3.38 ± 0.09 ^b^	3.23 ± 0.04 ^b^	3.86 ± 0.13 ^a^	3.50 ± 0.26 ^a,b^

^1^ L, linoleic; O, oleic; P, palmitic; S, stearic. ^2^ Equivalent carbon number (ECN) = carbon number − 2 × double bonds. Mean values with a different letters within a row are significantly different from each other at *p* < 0.05.

**Table 2:** Fatty acid distribution (% of total fatty acids) on the glycerol backbone of different types of camellia oils.

Fatty Acids	*C. oleifera*			*C. semiserrata*			*C. gauchowensis*		
	Total	Sn-2	Sn-1,3	Total	Sn-2	Sn-1,3	Total	Sn-2	Sn-1,3
C14:0	0.03 ± 0.00 ^B,c^	0.06 ± 0.00 ^A,a^	0.02 ± 0.00 ^C,d^	0.07 ± 0.01 ^B,a^	ND ^1^	0.10 ± 0.01 ^A,a^	0.05 ± 0.00 ^B,b^	ND	0.08 ± 0.00 ^A,c^
C16:0	9.51 ± 0.00 ^B,b^	4.85 ± 0.01 ^C,c^	11.83 ± 0.00 ^A,c^	8.53 ± 0.04 ^B,e^	1.30 ± 0.01 ^C,f^	12.15 ± 0.06 ^A,b^	11.52 ± 0.02 ^B,a^	2.26 ± 0.01 ^C,e^	16.15 ± 0.04 ^A,a^
C16:1	0.10 ± 0.01 ^B,b^	ND	0.15 ± 0.01 ^A,c^	0.09 ± 0.00 ^B,b^	ND	0.13 ± 0.00 ^A,d^	0.07 ± 0.00 ^B,c^	ND	0.10 ± 0.00 ^A,e^
C17:0	0.09 ± 0.00 ^B,a^	ND	0.14 ± 0.00 ^A,a^	0.08 ± 0.00 ^B,b^	ND	0.12 ± 0.00 ^A,b^	0.09 ± 0.00 ^B,a^	ND	0.13 ± 0.00 ^A,a^
C18:0	2.38 ± 0.01 ^B,c^	0.67 ± 0.00 ^C,a^	3.23 ± 0.01 ^A,c^	3.19 ± 0.03 ^B,a^	0.36 ± 0.01 ^C,b^	4.61 ± 0.05 ^A,a^	2.69 ± 0.01 ^B,b^	0.67 ± 0.01 ^C,a^	3.71 ± 0.02 ^A,b^
C18:1 n-9	76.64 ± 0.03 ^B,d^	81.15 ± 0.07 ^A,d^	74.38 ± 0.07 ^C,d^	77.99 ± 0.01 ^B,a^	86.85 ± 0.07 ^A,a^	73.57 ± 0.02 ^C,e^	75.00 ± 0.01 ^B,e^	81.70 ± 0.14 ^A,c^	71.65 ± 0.05 ^C,f^
C18:2 n-6	10.25 ± 0.01 ^B,c^	12.95 ± 0.07 ^A,b^	8.90 ± 0.02 ^C,b^	9.16 ± 0.02 ^B,f^	11.25 ± 0.07 ^A,d^	8.12 ± 0.07 ^C,d^	9.64 ± 0.01 ^B,e^	14.90 ± 0.14 ^A,a^	7.01 ± 0.09 ^C,e^
C20:0	0.09 ± 0.00 ^B,b^	0.03 ± 0.00 ^C,b^	0.12 ± 0.01 ^A,c^	0.10 ± 0.00 ^B,b^	ND	0.15 ± 0.01 ^A,b^	0.06 ± 0.00 ^B,d^	0.10 ± 0.01 ^A,a^	0.03 ± 0.01 ^C^ e
C20:1 n-9	0.52 ± 0.02 ^B,a,b^	ND	0.78 ± 0.04 ^A,a^	0.56 ± 0.01 ^B,a^	ND	0.84 ± 0.01 ^A,a^	0.52 ± 0.03 ^B,b^	ND	0.78 ± 0.05 ^A,a^
C18:3 n-3	0.32 ± 0.01 ^A,a^	0.32 ± 0.03 ^A,a^	0.32 ± 0.04 ^A,a,b^	0.13 ± 0.01 ^B,c^	0.18 ± 0.03 ^A,b^	0.11 ± 0.14 ^C,c^	0.30 ± 0.02 ^A,a^	0.33 ± 0.00 ^A,a^	0.29 ± 0.03 ^A,b^
C22:1 n-9	0.08 ± 0.00 ^B,b^	ND	0.12 ± 0.01 ^A,b^	0.09 ± 0.01 ^B,a^	ND	0.14 ± 0.01 ^A,a^	0.07 ± 0.00 ^B,b^	ND	0.11 ± 0.00 ^A,b^
SFA	12.10 ± 0.01 ^B,b^	5.61 ± 0.02 ^C,c^	15.34 ± 0.02 ^A,c^	11.97 ± 0.01 ^B,c^	1.66 ± 0.02 ^C,f^	17.12 ± 0.00 ^A,b^	14.41 ± 0.03 ^B,a^	3.03 ± 0.02 ^C,e^	20.10 ± 0.06 ^A,a^
MUFA	77.34 ± 0.01 ^B,d^	81.15 ± 0.07 ^A,d^	75.43 ± 0.05 ^C,d^	78.73 ± 0.00 ^B,a^	86.85 ± 0.07 ^A,a^	74.67 ± 0.03 ^C,e^	75.65 ± 0.02 ^B,e^	81.70 ± 0.14 ^A,c^	72.63 ± 0.11 ^C,f^
PUFA	10.57 ± 0.01 ^B,c^	13.27 ± 0.10 ^A,b^	9.21 ± 0.06 ^C,c^	9.30 ± 0.01 ^B,f^	11.43 ± 0.04 ^A,d^	8.23 ± 0.03 ^C,e^	9.94 ± 0.01 ^B,e^	15.23 ± 0.14 ^A,a^	7.29 ± 0.06 ^C,f^
**Fatty acids**	**Commercial *C. oleifera***			**Commercial *C. oleifera*-DAG (40%)**			**Commercial *C. oleifera*-DAG (80%)**		
	**Total**	**Sn-2**	**Sn-1,3**	**Total**	**Sn-2**	**Sn-1,3**	**Total**	**Sn-2**	**Sn-1,3**
C14:0	0.06 ± 0.00 ^B,a^	ND	0.09 ± 0.00 ^A,a,b^	0.06 ± 0.00 ^B,a^	ND	0.08 ± 0.00 ^A,b,c^	0.05 ± 0.00 ^B,b^	ND	0.08 ± 0.00 ^A,c^
C16:0	9.21 ± 0.01 ^B,c^	3.88 ± 0.19 ^C,d^	11.87 ± 0.09 ^A,c^	8.75 ± 0.01 ^B,d^	8.22 ± 0.07 ^C,b^	9.02 ± 0.04 ^A,d^	8.26 ± 0.01 ^B,f^	14.05 ± 0.07 ^A,a^	5.36 ± 0.02 ^C,e^
C16:1	0.14 ± 0.00 ^B,a^	ND	0.21 ± 0.00 ^A,a^	0.13 ± 0.00 ^B,a^	ND	0.20 ± 0.00 ^A,a,b^	0.13 ± 0.00 ^B,a^	ND	0.19 ± 0.00 ^A,b^
C17:0	0.07 ± 0.00 ^B,c^	ND	0.10 ± 0.00 ^A,c^	0.07 ± 0.00 ^B,c^	ND	0.10 ± 0.00 ^A,c,d^	0.06 ± 0.00 ^B,d^	ND	0.09 ± 0.00 ^A,e^
C18:0	2.00 ± 0.00 ^B,d^	ND	2.99 ± 0.00 ^A,d^	2.01 ± 0.00 ^B,d^	ND	3.01 ± 0.00 ^A,d^	2.01 ± 0.00 ^B,d^	ND	3.02 ± 0.00 ^A,d^
C18:1 n-9	77.65 ± 0.01 ^B,b^	83.00 ± 0.28 ^A,b^	74.98 ± 0.13 ^C,c^	77.64 ± 0.01 ^B,b^	78.60 ± 0.28 ^A,e^	77.17 ± 0.13 ^B,b^	77.60 ± 0.03 ^B,c^	73.60 ± 0.00 ^C,f^	79.60 ± 0.05 ^A,a^
C18:2 n-6	10.02 ± 0.00 ^B,d^	13.15 ± 0.07 ^A,b^	8.46 ± 0.04 ^C,c^	10.45 ± 0.00 ^B,b^	13.20 ± 0.28 ^A,b^	9.07 ± 0.14 ^C,b^	10.91 ± 0.07 ^B,a^	12.35 ± 0.07 ^A,c^	10.18 ± 0.06 ^C,a^
C20:0	0.07 ± 0.00 ^B,c^	ND	0.10 ± 0.00 ^A,d^	0.09 ± 0.00 ^B,b^	ND	0.13 ± 0.00 ^A,b^	0.11 ± 0.00 ^B,a^	ND	0.17 ± 0.00 ^A,a^
C20:1 n-9	0.52 ± 0.00 ^B,a,b^	ND	0.78 ± 0.00 ^A,a^	0.52 ± 0.00 ^B,a,b^	ND	0.78 ± 0.00 ^A,a^	0.53 ± 0.00 ^B,a,b^	ND	0.79 ± 0.00 ^A,a^
C18:3 n-3	0.23 ± 0.00 ^B,b^	ND	0.34 ± 0.00 ^A,a,b^	0.23 ± 0.00 ^B,b^	ND	0.35 ± 0.00 ^A,a^	0.24 ± 0.00 ^B,b^	ND	0.36 ± 0.00 ^A,a^
C22:1 n-9	0.04 ± 0.00 ^B,c^	ND	0.06 ± 0.00 ^A,d^	0.05 ± 0.00 ^B,c^	ND	0.07 ± 0.00 ^A,c,d^	0.05 ± 0.00 ^B,c^	ND	0.08 ± 0.00 ^A,c^
SFA	11.40 ± 0.01 ^B,d^	3.88 ± 0.19 ^C,d^	15.16 ± 0.08 ^A,c^	10.98 ± 0.01 ^B,e^	8.22 ± 0.07 ^C,b^	12.35 ± 0.04 ^A,d^	10.50 ± 0.01 ^B,f^	14.05 ± 0.07 ^A,a^	8.72 ± 0.02 ^C,e^
MUFA	78.35 ± 0.01 ^B,b^	83.00 ± 0.28 ^A,b^	76.03 ± 0.13 ^C,c^	78.35 ± 0.01 ^A,b,c^	78.60 ± 0.28 ^B,e^	78.22 ± 0.13 ^C,b^	78.31 ± 0.03 ^B,c^	73.60 ± 0.00 ^C,f^	80.66 ± 0.04 ^A,a^
PUFA	10.25 ± 0.00 ^B,d^	13.15 ± 0.07 ^A,b^	8.80 ± 0.04 ^C,d^	10.68 ± 0.00 ^B,b^	13.20 ± 0.28 ^A,b^	9.42 ± 0.14 ^C,b^	11.15 ± 0.07 ^B,b^	12.35 ± 0.07 ^A,c^	10.55 ± 0.06 ^C,a^

^1^ Not detected. Mean values with different letters are significantly different from each other at *p* < 0.05. Mean values with capital letters represent differences in the distribution of fatty acids of each camellia oil on the glycerol backbones; mean values with lowercase letters represent differences in the distribution of different types of camellia oil on their respective glycerol backbones.

## Data Availability

The original contributions presented in the study are included in the article/Supplementary Material, further inquiries can be directed to the corresponding author.

## Supplementary Materials

The following supporting information can be downloaded at: https://www.mdpi.com/article/10.3390/foods13182894/s1, Table S1: Volatile compounds of the different types of camellia oils (mg/kg). ^1^ Not detected. C.O, *C. oleifera*; C.S, *C. semiserrata*, C.G, *C. gauchowensis*, C-C.O, commercial *C. oleifera*; C-C.O-DAG (40%), commercial *C. oleifera* diacylglycerol oil (40%), and C-C.O-DAG (80%), commercial *C. oleifera* diacylglycerol oil (80%); Figure S1: A typical chromatogram of the glyceride distribution in DAG-enriched camellia oils. TAG, triacylglycerol; DAG, diacylglycerol; MAG, monoacylglycerol, and FFA, free fatty acid.
