# Supplementary material for: A Comparative Study on the Characteristics of Different Types of Camellia Oils Based on Triacylglycerol Species, Bioactive Components, Volatile Compounds, and Antioxidant Activity
Source: Foods. 2024 Sep 12;13(18):2894. doi: 10.3390/foods13182894 (PMC11431759; doi:10.3390/foods13182894)
Supplement: Supplementary file 1 [file foods-13-02894-s001.zip › foods-3175023-supplementary.pdf]

Table S1. Volatile compounds of the different types of camellia oils (mg/kg).

Compounds	*C.O*	*C.S*	C.G	C-*C.O*	C-*C.O*-DAG (40%)	C-*C.O*-DAG (80%)
Methyl Alcohol	0.08	0.04	0.06	ND[1]	ND	ND
Ethanol	0.14	0.47	6.59	0.11	1.16	0.87
1-Propanol	0.04	ND	0.17	ND	ND	ND
1-Butanol	1.17	0.38	1.36	0.08	ND	ND
1-Pentanol	0.14	0.14	0.14	0.05	0.02	0.02
2-Buten-1-ol	0.01	ND	ND	ND	ND	ND
1-Hexanol	0.63	0.09	0.41	ND	ND	ND
1-Octen-3-ol	0.06	ND	ND	ND	ND	ND
2,3-Butanediol	0.18	0.72	0.39	ND	ND	ND
1-Octanol	0.23	ND	0.12	ND	ND	ND
2,3-Butanediol	0.16	1.16	0.49	ND	ND	ND
(S)-(+)-1,2-Propanediol	0.01	0.01	0.02	ND	ND	ND
2-Propanol	0.01	0.03	ND	ND	ND	ND
Benzyl alcohol	0.03	ND	ND	ND	ND	ND
Phenylethyl Alcohol	0.28	0.15	0.22	ND	0.05	0.12
Maltol	0.01	ND	ND	ND	ND	ND
3-Phenylpropanol	0.01	ND	ND	ND	ND	ND
(S)-3-Ethyl-4-methylpentanol	ND	0.09	ND	ND	ND	ND
meso-Hydrobenzoin	ND	0.02	ND	0.01	ND	ND
Ethane-1,1-diol Dipropanoate	ND	ND	0.02	ND	ND	ND
1-Penten-3-ol	ND	ND	ND	0.04	0.17	0.24
4-Hydroxy-3-hexanone	ND	ND	ND	0.02	ND	ND
1,2-Ethanediol	ND	ND	ND	0.02	ND	ND
1,4-Butanediol	ND	ND	ND	0.01	ND	ND
(S)-(+)-3-Methyl-1-heptanol	ND	ND	ND	ND	0.02	0.02
Acetic acid	0.76	4.48	2.52	0.49	0.27	0.34
Propanoic acid	0.08	0.08	0.22	0.07	0.09	0.12
Butanoic acid	0.52	0.55	1.29	0.03	0.04	0.05
Pentanoic acid	0.09	0.07	0.04	0.04	0.04	0.04
2-Butenoic acid	0.49	ND	0.03	ND	ND	ND
Hexanoic acid	0.29	0.30	0.30	0.19	0.13	0.10
Heptanoic acid	0.02	0.02	0.02	0.03	0.01	0.01
Octanoic acid	0.04	0.06	0.14	ND	0.01	0.01
Nonanoic acid	0.02	ND	0.02	ND	0.01	0.02
Formic acid	0.50	ND	0.19	ND	0.13	0.09
2-Methylbutanoic anhydride	ND	ND	ND	ND	0.01	0.01
2-Propenoic acid	ND	0.05	0.02	ND	ND	ND
Ethyl Acetate	0.02	0.14	1.03	0.05	0.03	0.01
Ethyl tiglate	1.06	ND	ND	ND	ND	ND
n-Caproic acid vinyl ester	0.01	ND	ND	ND	ND	ND

Allyl Isothiocyanate	0.01	0.08	0.13	ND	ND	ND
2(3H)-Furanone	0.02	0.01	0.14	ND	ND	ND
Butyrolactone	0.04	0.41	ND	ND	ND	ND
Dihydro-3-methylene-5-methyl-2-furanone	0.02	ND	ND	ND	ND	ND
Vinyl trans-cinnamate	ND	ND	0.09	ND	ND	ND
1-Methoxy-2-propyl acetate	ND	0.37	ND	ND	ND	ND
Hydrogen isocyanate	ND	ND	0.01	ND	ND	ND
n-Propyl acetate	ND	ND	0.01	0.02	ND	ND
Ethyl 6-methylpyridine-2-carboxylate	ND	ND	0.03	ND	ND	ND
sec-Butyl acetate	ND	ND	ND	0.05	ND	ND
Methyl methacrylate	ND	ND	ND	ND	ND	0.01
Butanedioic acid	ND	ND	ND	ND	0.04	ND
Pentanedioic acid	ND	ND	ND	ND	0.04	ND
Butanal	0.25	ND	0.20	0.05	0.02	0.03
Hexanal	1.11	1.07	0.06	0.60	0.62	0.89
Heptanal	0.29	ND	1.41	ND	0.03	0.04
Octanal	0.33	0.20	0.22	ND	0.04	0.04
2-Heptenal	0.05	ND	0.03	ND	ND	ND
Nonanal	1.77	0.21	ND	ND	0.04	0.06
5-Ethylcyclopent-1-enecarboxaldehyde	0.01	ND	ND	ND	ND	ND
3-Furaldehyde	0.04	ND	0.01	ND	ND	ND
Benzaldehyde	0.29	0.25	0.27	ND	ND	ND
Benzeneacetaldehyde	1.23	0.17	0.23	ND	ND	ND
Pentanal	ND	0.05	ND	0.24	0.19	0.16
2-Butenal	ND	0.12	ND	ND	0.04	0.06
Propanal	ND	ND	ND	0.10	ND	ND
2-Propenal	ND	ND	ND	ND	0.06	0.06
2-Nonenal	ND	ND	ND	ND	0.03	0.05
2-Decenal	ND	ND	ND	ND	0.02	0.03
Acetone	0.04	ND	ND	0.04	ND	ND
2-Butanone	0.01	0.01	ND	0.01	0.03	0.06
2-Pentanone	0.10	ND	0.13	ND	ND	ND
2-Octen-4-one	0.02	ND	ND	ND	ND	ND
2-Heptanone	0.04	ND	ND	ND	ND	ND
2,4-Azetidinedione	0.01	ND	ND	ND	ND	ND
Acetoin	0.06	0.41	0.15	ND	ND	ND
2-Propanone	0.02	0.01	0.01	ND	ND	ND
5-Hepten-2-one	0.01	ND	ND	ND	ND	ND
2,3-Butanedione	ND	0.13	ND	ND	ND	ND
2,3-Pentanedione	ND	0.01	0.01	0.02	0.01	0.01
3-Heptanone	ND	ND	ND	0.35	ND	ND

1-Pentanone	ND	ND	ND	0.05	ND	ND
1-Hydroxy-2-butanone	ND	ND	ND	0.03	0.02	0.02
Toluene	0.02	0.22	0.13	0.37	0.04	0.05
Styrene	0.50	0.12	0.14	ND	ND	ND
Phenol	ND	0.01	0.01	ND	ND	ND
Benzene	0.01	0.19	0.12	0.17	0.14	0.26
Octane	1.85	0.01	0.24	0.02	0.65	0.57
Hexane	0.03	0.16	ND	ND	ND	ND
Nonane	0.08	ND	ND	ND	ND	ND
Decane	0.11	0.10	0.19	ND	ND	ND
Cyclopropane	0.01	ND	ND	ND	ND	ND
Heptane	0.01	0.02	0.25	ND	ND	ND
Bicyclo[3.1.0]hex-2-ene	ND	ND	0.10	ND	ND	ND
1-Butene	0.02	ND	ND	ND	ND	ND
D-Limonene	0.02	0.68	1.11	0.12	ND	ND
Cyclohexene	0.01	ND	ND	ND	ND	ND
Cyclopentane	0.03	0.01	0.01	ND	ND	ND
3-Ethyl-3-methylheptane	ND	0.02	ND	ND	ND	ND
Undecane	ND	0.02	0.02	ND	ND	ND
1-Pentene	ND	0.14	ND	0.03	ND	ND
Neopentane	ND	ND	0.02	ND	ND	ND
Trichloromethane	ND	ND	0.21	ND	ND	0.03
Pentane	ND	ND	0.04	ND	ND	ND
β-Myrcene	ND	ND	0.29	ND	ND	ND
Cycloheptane	ND	ND	0.58	ND	ND	ND
1-Isopropylcyclohex-1-ene	ND	ND	ND	ND	0.18	0.36
Furan	0.26	ND	ND	ND	0.20	0.35
Disulfide	0.01	ND	ND	ND	ND	ND
Tetrahydrofuran	ND	0.14	0.14	ND	ND	ND
N-Methyl methacrylamide	ND	0.05	ND	ND	ND	ND
Formamide	ND	ND	ND	0.02	ND	ND

[1] Not detected. *C.O, C. oleifera; C.S, C. semiserrata, C.G, C. gauchowensis*, C-*C.O*, commercial *C. oleifera;* C-*C.O*-DAG (40%), commercial *C. oleifera* diacylglycerol oil (40%), and C-*C.O*-DAG (80%), commercial *C. oleifera* diacylglycerol oil (80%).

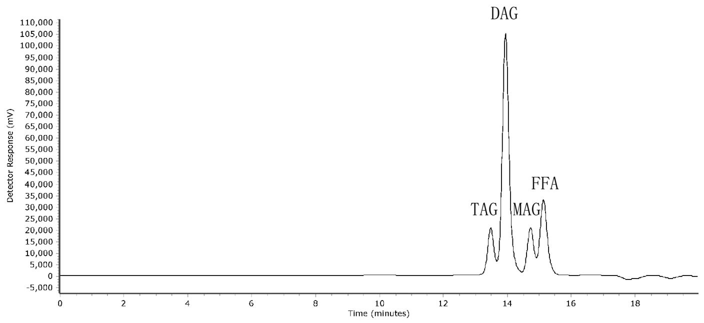

Figure S1. A typical chromatogram of the glyceride distribution in DAG-enriched camellia oils. TAG, triacylglycerol; DAG, diacylglycerol; MAG, monoacylglycerol, and FFA, free fatty acid.
